# A comparison between traditional and measurement-error growth models for weakfish *Cynoscion regalis*

**DOI:** 10.7717/peerj.2431

**Published:** 2016-09-21

**Authors:** Joshua Hatch, Yan Jiao

**Affiliations:** 1 Integrated Statistics, Inc., Woods Hole, Massachusetts, United States; 2 Department of Fish and Wildlife Conservation, Virginia Polytechnic Institute and State University (Virginia Tech), Blacksburg, Virginia, United States

**Keywords:** Bayesian, Weakfish, Growth, Length-at-age, Ageing error

## Abstract

Inferring growth for aquatic species is dependent upon accurate descriptions of age-length relationships, which may be degraded by measurement error in observed ages. Ageing error arises from biased and/or imprecise age determinations as a consequence of misinterpretation by readers or inability of ageing structures to accurately reflect true age. A Bayesian errors-in-variables (EIV) approach (i.e., measurement-error modeling) can account for ageing uncertainty during nonlinear growth curve estimation by allowing observed ages to be parametrically modeled as random deviates. Information on the latent age composition then comes from the specified prior distribution, which represents the true age structure of the sampled fish population. In this study, weakfish growth was modeled by means of traditional and measurement-error von Bertalanffy growth curves using otolith- or scale-estimated ages. Age determinations were assumed to be log-normally distributed, thereby incorporating multiplicative error with respect to ageing uncertainty. The prior distribution for true age was assumed to be uniformly distributed between ±4 of the observed age (yr) for each individual. Measurement-error growth models described weakfish that reached larger sizes but at slower rates, with median length-at-age being overestimated by traditional growth curves for the observed age range. In addition, measurement-error models produced slightly narrower credible intervals for parameters of the von Bertalanffy growth function, which may be an artifact of the specified prior distributions. Subjectivity is always apparent in the ageing of fishes and it is recommended that measurement-error growth models be used in conjunction with otolith-estimated ages to accurately capture the age-length relationship that is subsequently used in fisheries stock assessment and management.

## Introduction

Effects of measurement error in solving nonlinear models have been well documented (Carroll et al., 2006), causing bias in parameter estimates (Solow, 1998; Jiao, Reid & Nudds, 2006; Biggs, Carpenter & Brock, 2009; Heery & Berkson, 2009), confounding relationships among covariates (Walters & Ludwig, 1981; Gustafson, 2003), and exaggerating model selection uncertainty (Punt et al., 2008; Biggs, Carpenter & Brock, 2009). Of particular concern is the role observation error plays in nonlinear growth curve estimation, as age-length relationships play a key role in eliciting biological reference points from age-structured stock assessment models. While several methods have been constructed to account for gear selectivity and variable length-at-age in fitting nonlinear growth curves (Sainsbury, 1980; Pilling, Kirkwood & Walker, 2002; Taylor, Walters & Martell, 2005; He & Bence, 2007; Alós et al., 2010; Jiao et al., 2010), relatively few approaches have been developed to incorporate ageing error when inferring growth for aquatic species (Kimura, 2000; Cope & Punt, 2007; Schwarz & Runge, 2009).

Ageing error is largely determined through multiple age reads of the same individual, with relative bias and imprecision being evaluated graphically through age-bias plots and/or various age-discrimination statistics (Chang, 1982; Campana, Annand & McMillan, 1995; Campana, 2001). If age validation data are available, then known biases can be corrected for during the model fitting process by calibrating observed ages to reflect true age estimates (Schwarz & Runge, 2009). Unfortunately, the majority of age-length data sets used in fisheries stock assessment comprise a single age and length measurement per individual with true age being unknown (Cope & Punt, 2007; Punt et al., 2008). A single age read per individual complicates the parameter estimation procedure, as traditional methods for correcting age misclassification require an estimate of the ageing error variance, necessitating multiple age reads per individual and/or that observed ages are randomly distributed around the latent variable of true age (Cook & Stefanski, 1994; Cope & Punt, 2007; Punt et al., 2008). As a consequence, most growth investigations assume ageing error is negligible or relatively non-influential, with respect to process noise, in describing the age-length relationship (Pondella et al., 2001; Harris et al., 2007). Ignoring ageing error may be an unreasonable approach, as conventional methods tend to underestimate the uncertainty in parameter values, with respect to error in both the dependent and independent variables, leading to overconfidence in the description of growth and subsequent management decisions derived from growth curve analyses (Clark, 1991).

Fisheries scientists have long recognized that most independent variables necessary for stock assessments are measured with non-negligible uncertainty, although most attention has been spent on estimating the degree of bias in parameter estimates instead of attenuating error through increased model complexity (Hilborn & Walters, 1992). Recent advances in computational techniques have led to increased utilization of measurement-error models that allow for uncertainty in both the dependent and independent variables (Clark, 2005; Jiao, Reid & Nudds, 2006), although it is still necessary to understand the tradeoffs between model articulation and descriptive accuracy (Costanza & Sklar, 1985; Clark, 2005; Biggs, Carpenter & Brock, 2009). A Bayesian approach can allow for stochasticity at multiple levels within a hierarchically structured framework for nonlinear regression (as can a frequentist approach), with presumed understanding of the independent variable’s distribution (i.e., true age) coming from the specified prior (Clark, 2007). Hence, Bayesian errors-in-variables (EIV) (i.e., measurement-error) models allow for the fitting of nonlinear growth curves when the ageing error distribution is unknown or inestimable using contemporary methods (i.e., one age read per individual).

Variability in age estimates for individual fish could be a consequence of misinterpretation by readers of ageing structures (e.g., scales and otoliths) or inability of ageing structures to accurately record growth sequence information (Neilson, 1992; Campana, 2001). While most calcified structures have the potential to provide accurate estimates of age (Campana, 2001), subjectivity is always apparent in the production ageing process undertaken for fisheries stock assessments (Kimura & Lyons, 1991; Heifetz et al., 1998; Morison, Robertson & Smith, 1998; Buckmeier, 2002). Two of the most commonly used hard parts in the assignment of age to individual fish include otoliths and scales (Hilborn & Walters, 1992), with the general understanding that otoliths provide more accurate and precise age estimates compared to scale-estimated ages (Lowerre-Barbieri, Chittenden & Barbieri, 1995; Maceina et al., 2007). However, various sources of error still confound the assignment of age to individual fish for otolith-estimated ages (Neilson, 1992; Pepin, Dower & Benoît, 2001) and incorporation of measurement error into nonlinear growth curve analysis is still prudent.

Weakfish *Cynoscion regalis* are a marine finfish found along the eastern coast of the United States (US), ranging from Massachusetts to Florida (Shepherd & Grimes, 1983). Historically, weakfish have supported important commercial and recreational fisheries along the US Northwest Atlantic (Nye, Targett & Helser, 2008), with relatively low landings in recent years as a result of management measures and low stock abundance (Northeast Fisheries Science Center, 2009). While several studies have investigated the age and growth of weakfish at various spatial and temporal scales (Seagraves, 1981; Shepherd & Grimes, 1983; Hawkins, 1988; Villoso, 1989; Lowerre-Barbieri, Chittenden & Barbieri, 1995), the effects of ageing error on describing the age-length relationship are largely unknown (Northeast Fisheries Science Center, 2009). The goal of this study was to evaluate and compare traditional and measurement-error growth models for weakfish *Cynoscion regalis* using otolith- or scale-estimated ages incorporating unbiased ageing error.

## Materials and Methods

### Data

**Figure 1:**
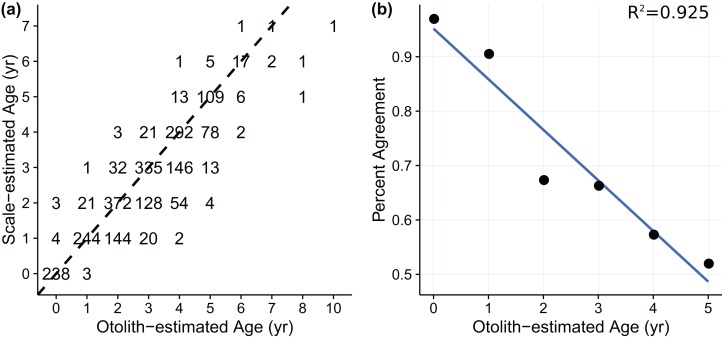
(A) age-bias plot for weakfish *Cynosicon regalis* using otolith-estimated and scale-estimated ages obtained from Wenner & Gregory (2000). Numbers correspond to sample size. Dotted line indicates 1:1 agreement between ototlith- and scale-estimated age. (B) percent agreement between otolith- and scale-estimated ages as a function of otolith-estimated age for weakfish *Cynoscion regalis*. Only ages 0–5 were used for comparison due to limited sample size of older individuals. Solid line indicates general trend.

Age-length data for weakfish *Cynoscion regalis* were obtained from Wenner & Gregory (2000), with age for the same individual being estimated from sagittal otolith and scale readings. The otolith-scale age comparison database comprised 2,318 weakfish caught intermittently from five states (i.e., New York, Delaware, Maryland, Virginia, and North Carolina) for years 1989, 1992, 1995, and 1996 (Table 1). Individuals were pooled across states and years to fit von Bertalanffy and measurement-error von Bertalanffy growth curves using otolith- or scale-estimated ages. An age-bias plot (Fig. 1A) indicated ageing uncertainty for weakfish, with scale readings tending toward younger age estimates compared to otolith-estimated ages. Also, percent agreement between ageing structures declined with age, suggesting error in the ability of readers to consistently discern age for older fish (i.e., multiplicative ageing uncertainty) (Fig. 1B).

**Table 1:** Summary of weakfish *Cynoscion regalis* age-length data used in constructing traditional and measurement-error von Bertalanffy growth models (Wenner & Gregory, 2000).

	1989	1992	1995	1996	Total
New York	0	0	114	0	114
Delaware	0	0	1,139	150	1,289
Maryland	0	0	0	95	95
Virginia	83	74	0	0	157
North Carolina	0	0	142	521	663
Total	83	74	1,395	766	2,318

### Nonlinear growth models

The von Bertalanffy growth function has a long history in fisheries science and has been used extensively to describe fish growth (i.e., length and weight) as a function of age (Haddon, 2001). Despite criticisms (Roff, 1980), the von Bertalanffy growth curve has been advocated as an appropriate growth model because of its ability to capture observed trends between length and age for a variety of fish species (Chen, Jackson & Harvey, 1992). A recent stock assessment modeled weakfish growth using a von Bertalanffy growth function (Northeast Fisheries Science Center, 2009) that can be written as:

(1) $${L_i} = {L_\infty }\left({1-{e^{\left({-k\left({{{t'\!}_i}-{t_o}} \right)} \right)}}} \right) \cdot {e^{{\varepsilon _i}}}$$

where *L_i_* is the length-at-age for the *i*th individual, *L_∞_* is the asymptotic length, *k* is the Brody growth coefficient, *t_o_* is the hypothetical length at age 0, and ${t'_i}$ is the observed age for the *i*th individual. Error *ε_i_* is assumed to be independent and normally distributed with mean 0 and variance $\sigma _L^2$.

Extending the von Bertalanffy growth model to incorporate measurement error is relatively straight forward, and can be written as:

(2) $$\eqalign{&{L_i} = {L_\infty }\left({1-{e^{\left({-k\left({{t_i}-{t_o}} \right)} \right)}}} \right) \cdot {e^{{\varepsilon _i}}} \cr & {{t'_i}} = {t_i} \cdot {e^{{\varepsilon _i}}}} $$

where *t_i_* is the true age for the *i*th individual. The logarithm of observed age ${\rm log_e}\left({{{t'}_i}} \right)$ is assumed to be independent and normally distributed with mean log_e_(*t_i_*) and variance $\sigma _A^2$. In order to facilitate the use of a log-normal distribution for observed ages, a small constant (i.e., 10E-05) was added to age-0 individuals during model fitting.

### Statistical estimator

A Bayesian estimator was used to construct the joint posterior probability distribution for parameters in the von Bertalanffy and measurement-error von Bertalanffy growth curves. The full conditional distribution for the traditional von Bertalanffy growth model is:

(3) $$p\left({{L_\infty },\;k,\,{t_o},\,\sigma _L^2|\;{\bf{\it L}}} \right) \propto \;\mathop \prod \limits_{i = 1}^n {\cal L}\left({{L_i}\;|\;{L_\infty },\;k,\,{t_o},\,\sigma _L^2} \right) \times \pi \left({\sigma _L^2} \right)\pi \left({{L_\infty }} \right)\pi \left(k \right)\pi \left({{t_o}} \right)$$

While the full conditional distribution for the measurement-error von Bertalanffy growth model is:

(4) $$\eqalign{p\left({{L_\infty },\;k,\,{t_o},\,\sigma _L^2,\,\sigma _A^{2\;},\,{\bf{\it t}}|\;{\bf{\it L}}} \right)\; \propto \mathop \prod \limits_{i = 1}^n {\cal L}\left({{L_i}\;|\;{L_\infty },\;k,\,{t_o},\,\sigma _L^2,\,{t_i}} \right){\cal L}\left({{{t'_i}}|\;{t_i},\,\sigma _A^2} \right) \times \pi \left({\sigma _L^2} \right)\cr\pi \left({\sigma _A^2} \right)\pi \left({{L_\infty }} \right)\pi \left(k \right)\pi \left({{t_o}} \right)\pi \left({{t_i}} \right)}$$

where *p*(·) denotes the posterior probability, ${\cal L}\left(\cdot \right)$ denotes the likelihood function, and $\pi \left(\cdot \right)$ denotes the prior distribution.

As shown in Eq. (4), observed lengths (*L_i_*) are conditionally independent of observed ages (${t'_i}$), with the majority of information about true age (*t_i_*) coming from the prior. Observed length will also inform the estimation of true age through feedback of the likelihoods on the joint posterior distribution, with length often assumed to be a loose proxy for age (e.g., age-length keys). Essentially, measurement-error growth models work to pull observations closer to the median length-at-age, suggesting the need for an informative prior on true age when multiple age determinations are unavailable. If an informative prior on true age is unjustifiable, then multiple age determinations will be necessary to estimate the ageing-error variance(s) or a reference collection will be required, in which true age for a set of individuals is known, so that validation data can help calibrate the model during estimation.

Prior distributions were constructed around historic estimates of weakfish growth, thereby encompassing biological relevancy (Seagraves, 1981; Shepherd & Grimes, 1983; Hawkins, 1988; Villoso, 1989; Lowerre-Barbieri, Chittenden & Barbieri, 1995). Age validation data were unavailable and consequently the latent variable of true age was assumed to follow a uniform distribution, with the lower and upper bound being defined by ±4 of the observed age for each individual, as the largest difference between otolith- and scale-estimated ages was three years (Fig. 1A). The weakfish age-length dataset was characterized by a lack of older, larger-sized individuals compared to the most recent investigation of age and growth (Lowerre-Barbieri, Chittenden & Barbieri, 1995). Changes in weakfish age- and size-structure are most likely a culmination of several factors, including: residual effects of excessive fishing mortality (Northeast Fisheries Science Center, 2009), gear selectivity, and seasonal variation in spatial distribution as a result of differential migration by size (Lowerre-Barbieri, Chittenden & Barbieri, 1995). In order to avoid inflated estimates of asymptotic length (*L_∞_*) and consequent underestimation of the Brody growth coefficient (*k*), posterior values for *L_∞_* were bounded by the specified prior distribution. A summary of prior distributions can be found in Table 2.

**Table 2:** Parameter estimates from traditional von Bertalanffy (VBGF) and measurement-error (MEVB) von Bertalanffy growth models using otolith-estimated and scale-estimated ages (i.e., M1–M4, see Table 3), including posterior mean, standard deviation (S.D.), and gelman and rubin diagnostic (R^2^).

Model	Parameters	Prior	Otolith			Scale		
			Mean	SD	R^2^	Mean	SD	R^2^
VBGF	*L_∞_*	U(300,1200)	1,177.780	21.982	1.02	1,179.558	19.132	1.00
	*k*	U(0,1)	0.068	0.002	1.03	0.076	0.002	1.00
	*t_o_*	U(−3,1)	−2.347	0.054	1.00	−2.116	0.045	1.00
	*σ_L_*	U(0.0001,10)	0.190	0.003	1.00	0.180	0.003	1.00
MEVB	*L_∞_*	U(300,1200)	1,187.649	11.840	1.00	1,187.139	12.671	1.00
	*k*	U(0,1)	0.062	0.001	1.00	0.068	0.001	1.00
	*t_o_*	U(−3,1)	−2.596	0.053	1.00	−2.359	0.047	1.00
	*σ_L_*	U(0.0001,10)	0.153	0.004	1.00	0.142	0.003	1.00
	*σ_A_*	U(0.0001,10)	0.275	0.010	1.00	0.281	0.010	1.00

All models were run with three Markov chains for 100,000 simulations per chain using the software packages WinBUGS version 1.4.3 and R version 2.13.1. Convergence of the Markov chains to the stationary distribution was determined by monitoring trace plots and computing Gelman and Rubin diagnostics. The first 50,000 iterations from each chain were discarded to allow for adequate burn-in and a thinning interval of five was used to reduce autocorrelation among iterative samples and improve computational efficiency. A total number of 30,000 iterations were used to summarize the posterior distribution for each model.

### Model selection criteria

Growth is a vital component in discerning the population dynamics of fishes and modeling age-length relationships requires the ability to effectively compare and discriminate among alternative hypotheses that represent biological realism. In this study, model checking and discrimination were conducted using posterior predictive *p*-values and deviance information criterion (DIC), respectively. While DIC has the potential to identify correct model structure for catch-at-age analyses (Wilberg & Bence, 2008), its ability to select preferred models in an EIV context is less clear (Spiegelhalter et al., 2002; Celeux et al., 2006). To circumvent this issue, posterior predictive model checks and model discrimination statistics were used in an effort to corroborate anecdotal beliefs regarding the applicability of measurement-error models during nonlinear growth curve analyses.

#### Posterior predictive *p*-values

Posterior predictive *p*-values were used to conduct posterior predictive model checks in evaluating the ability of posited models to replicate data similar to that observed. Generally, a discrepancy statistic is used to assess model goodness-of-fit based on observed data and the posterior predictive distribution, where the posterior predictive distribution is defined as:

(5) $$p\left({{y^{rep}}{\rm{|}}y} \right) = \int\! p\left({{y^{rep}}{\rm{|}}\theta } \right)p(\theta |y)d\theta $$

where *p*(*y^rep^|θ*) is the data distribution for the replicated observations *y^rep^* and *p(θ|y)* is the posterior distribution for the unknown parameter vector *θ* given the observed data *y*. The discrepancy measure utilized in this study was the Bayesian residual sum of squares (Gelman, Meng & Stern, 1996), which can be written as:

(6) $${\chi ^2}\left({y;\theta } \right) = \mathop \sum \limits_{i = 1}^n {{{{\left({{y_i}-E\left({{y_i}{\rm{|}}\theta } \right)} \right)}^2}} \over {Var\left({{y_i}{\rm{|}}\theta } \right)}}$$

where *E*(·) is the expectation, *Var*(·) is the variance, and *y_i_* is the *i*th observation of the data *y* or simulated data *y^rep^*. The posterior predictive *p*-value, then, is simply the proportion of times χ^2^(*y^rep^*; θ) ≥ χ^2^(*y*; θ). The closer the posterior predictive *p*-value is to 0.50, the more adequate the model is at replicating data similar to that observed.

#### Deviance information criterion

DIC was used to compare model goodness-of-fit, as measurement-error models are hierarchically structured and the number of parameters is difficult to enumerate (Spiegelhalter et al., 2002; Ward, 2008; Wilberg & Bence, 2008). Like other information-theoretic approaches, DIC penalizes overparamaterization and descriptive accuracy in order to select effective models with high explanatory power. DIC can be written as:

(7) $$ \eqalign{& DIC = \bar D + {p_D} \cr  &{p_D} = \bar D + D\left({\bar \theta } \right) \cr  & D\left(\cdot \right) =-2{\rm{log}}\left({{\cal L}\left({y{\rm{|}}\theta } \right)} \right)} $$

where *D*(·) is the deviance defined as −2 times the log-likelihood of the data *y* given the unknown parameter vector *θ*, $\bar D$ is the posterior mean of the deviance, $D\left({\bar \theta } \right)$ is the deviance evaluated at the posterior mean of *θ*, and *p_D_* is the effective number of parameters in the Bayesian model as formulated by Spiegelhalter et al. (2002). While Celeux et al. (2006) recommend alternatives to this definition of DIC for missing-data models, of which EIV regression is a subset; our approach is to use the most commonly encountered form within fisheries science.

### Simulation study

**Figure 2:**
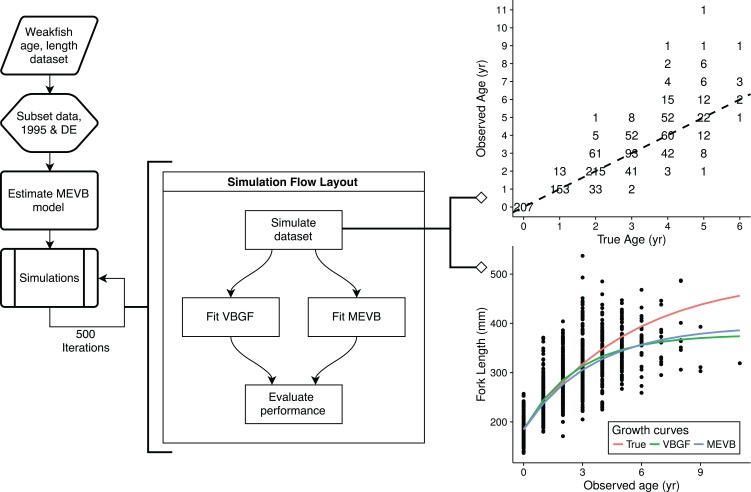
A flowchart for the simulation study to evaluate the performance of the traditional (VBGF) and measurement error (MEVB) von Bertalanffy growth models using 1 or 2 age reads. An example of the simulated datasets generated during the simulation is also presented. The example dataset shown in the upper, right-hand corner displays an age-bias plot where numbers correspond to sample size and the dotted line indicates 1:1 agreement between the true and observed age.

To explore the advantages of accounting for ageing error during nonlinear growth curve analyses, we ran simulations that emulated weakfish growth fitting both VBGF and MEVB models to simulated datasets using 1 or 2 age reads. In order to avoid modeling artifacts, we subsetted the original data to only include records collected in 1995 by the state of Delaware, when size truncation was less pronounced. The MEVB model was then fitted to the subsetted dataset using otolith-estimated ages, with the posterior averages of the estimated parameters (i.e., ***t***, *L_∞_*, *k*, *t_0_*, *σ_A_*, and *σ_L_*) then serving as the true, known values for the simulation. A workflow for the simulation is presented in Fig. 2. Accuracy of the two models was then investigated using the mean absolute error (MAE) and root mean square error (RMSE) statistics,

(8) $${\rm{MAE}} = {1 \over n}\mathop \sum \limits_i \left| {\widehat {{\theta _i}}-\theta } \right|$$

(9) $${\rm {RMSE}} = \sqrt {{1 \over n}\mathop \sum \limits_{\bf{\it i}} {{\left({\widehat {{\theta _i}}-\theta } \right)}^2}} $$

where *n* is the number of simulations (*n* = 500), $\widehat {{\theta _i}}$ is the estimated parameter at the *i*th simulation, and ***θ*** is the true, known value used to generate the simulated datasets.

## Results

### Model discrimination

**Figure 3:**
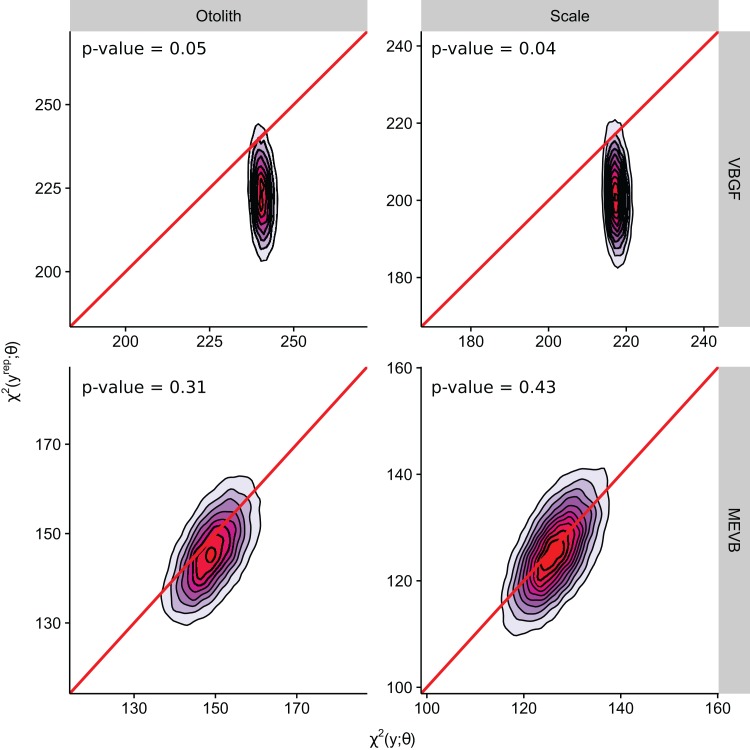
Two-dimensional density estimation of discrepancy statistics used in computing Bayesian posterior predictive *p*-values for models M1–M4 (see Table 3). Solid line indicates zero difference between the discrepancy statistic evaluated at the observed and replicated data. Colors represent low (blue) to high (red) densities.

According to the DIC statistic, traditional von Bertalanffy growth curves outperformed measurement-error growth models for both otolith- and scale-estimated ages (Table 3). Alternatively, posterior predictive *p*-values for measurement-error growth curves were substantially closer to 0.50 (Table 3; Fig. 3), suggesting improved adequacy of EIV models to reflect observed trends in the age-length relationship for weakfish. All growth curves considered in this study, however, had posterior predictive *p*-values < 0.50, possibly suggesting underparameterization in the ability of formulated models to partition the overall variance to its respective sources (i.e., variability in age or length). It is also apparent that inclusion of measurement error resulted in correlation between the observed and predicted lengths (Fig. 3), as estimation of true age is being informed, in part, by observed length. Nonetheless, predictive approaches to model comparison may be beneficial for EIV regression, as the utility of information-theoretic-based methods for measurement-error model selection are still circumstantial (Jiao, Reid & Smith, 2009).

**Table 3:** Model comparison of traditional (VBGF) and measurement-error (MEVB) von Bertalanffy growth models using posterior predictive *p*-values and deviance information criterion (DIC).

Scenario	Data	Model	*p*-value	$ {\bar {\bf{\it D}}}$	pD	DIC
M1	Otolith	VBGF	0.05	25,662	3	25,665
M3		MEVB	0.31	24,813	2,112	26,935
M2	Scale	VBGF	0.04	25,419	3	25,421
M4		MEVB	0.43	24,145	2,136	26,281

### von Bertalanffy growth curve parameters

**Figure 4:**
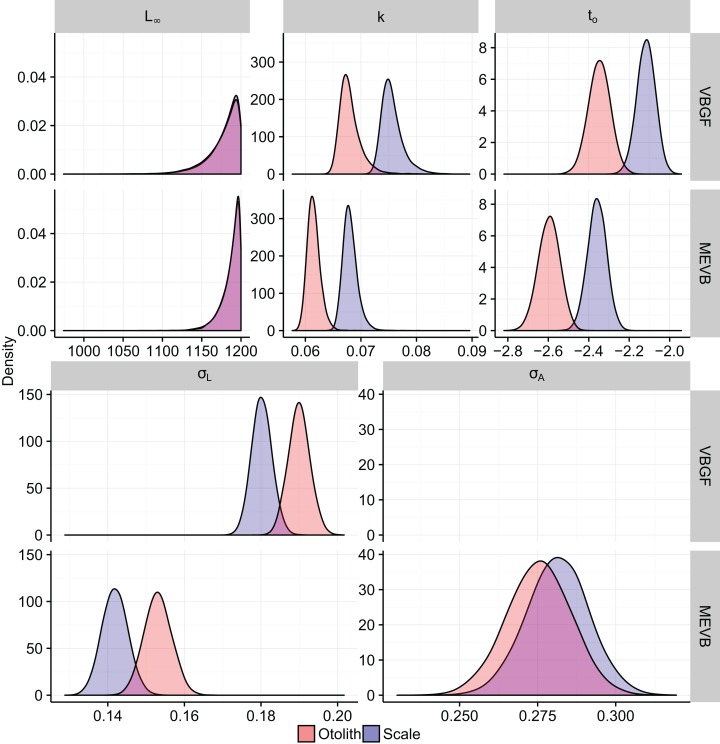
Marginal posterior distributions of the traditional (VBGF) and measurement-error (MEVB) von Bertalanffy growth curve parameters.

**Figure 5:**
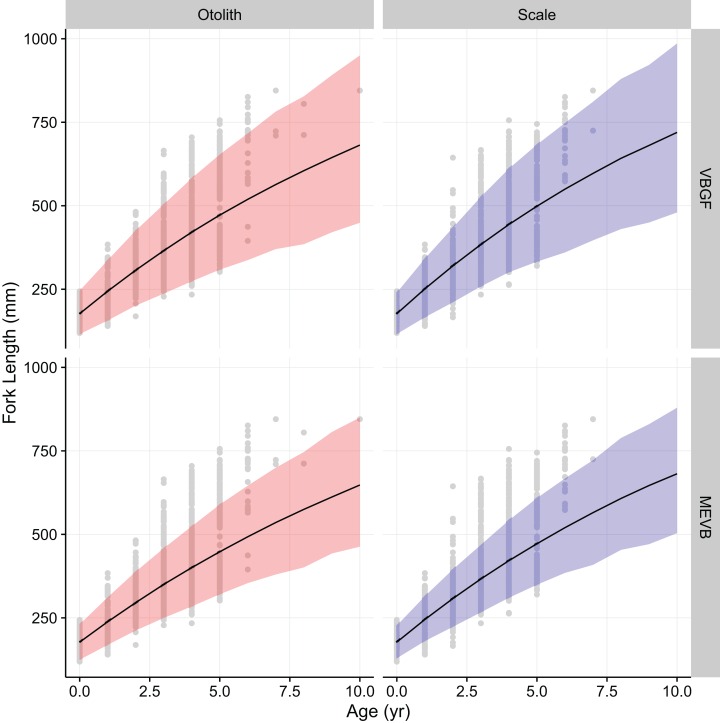
von Bertalanffy growth curves using otolith-estimated and scale-estimated ages. Solid lines correspond to median values of length-at-age from traditional (VBGF) and measurement-error (MEVB) von Bertalanffy growth models. The light-shaded regions correspond to 95% prediction intervals. Circles denote the observed data.

Growth models considering ageing error resulted in higher posterior mean values for *L_∞_* and *t_o_* (Table 2; Fig. 4), while producing lower posterior mean values for *k* and *σ_L_* (Table 2; Fig. 4). The posterior mean value for *σ_A_* was higher for scale-estimated ages, although there was substantial overlap between marginal posterior distributions (Fig. 4). Truncation of the joint posterior distribution for *L_∞_* and *k* was expected, as specified priors were used to constrain posterior draws to biologically reasonable values. The age-length data for weakfish fail to accurately capture the asymptotic length, leading to unrealistic estimates that are based on extrapolation of the age-length trend (Knight, 1968). As a consequence, measurement-error models demonstrated growth patterns where weakfish grew to reach larger sizes but at slower rates, with traditional von Bertalanffy growth curves overestimating median length-at-age for the observed age range (Fig. 5).

In addition, 95% prediction intervals were wider for traditional von Bertalanffy growth models, compared to their measurement-error analogs (Fig. 5). This is not surprising given that measurement-error models produced lower estimates for variability in predicted lengths (i.e., *σ_L_*), as some of the total variance gets partitioned out for ageing error. Generally, measurement-error growth models produced slightly narrower credible intervals for parameters of the von Bertalanffy growth function, with less difference between posterior mean values for biologically relevant parameter estimates using the different ageing structures (Table 2; Fig. 4).

### Simulations

The MAE and RMSE statistics were similar, with only the RMSE being reported for brevity (see Table 4). Results from the simulation study found that the estimated posterior means of the parameters from the MEVB growth models were closer to the “true” values, on average, when ageing error was present (Table 4). This suggests DIC may be inadequate at recommending growth models within EIV contexts, and researchers should pursue other model discrimination statistics when trying to confirm the utility of incorporating ageing uncertainty into growth curve analyses. The simulations also confirmed the intuition gained by using posterior predictive *p*-values, in that measurement-error models are more adequate at describing growth patterns for weakfish by lessening the degrading effects of ageing error. It is also apparent that multiple age reads can improve estimation of the ageing uncertainty, with simulations also showing that estimated variability in predicted lengths was more biased when ageing error was not considered (Table 4).

**Table 4:** Root mean squared error (RMSE) for parameters of the traditional (VBGF) and measurement error (MEVB) von Bertalanffy growth models using 1 or 2 age reads in the simulation study.

Model	Age reads	Parameters	True values	RMSE
VBGF	1	L_∞_	496.983	113.960
		k	0.185	0.183
		t_o_	−2.507	0.701
		σ_L_	0.121	0.016
MEVB	1	L_∞_	496.983	96.570
		k	0.185	0.103
		t_o_	−2.507	0.338
		σ_L_	0.121	0.005
		σ_A_	0.261	0.108
MEVB	2	L_∞_	496.983	77.461
		k	0.185	0.082
		t_o_	−2.507	0.315
		σ_L_	0.121	0.004
		σ_A_	0.261	0.096

## Discussion

Conceptually, the EIV approach is trying to correct the misallocation of younger, smaller-sized individuals to older age classes and older, larger-sized individuals to younger age classes, resulting in higher estimates for *L_∞_* and lower estimates for *k*. While the biological association between maximum size and the Brody growth coefficient may be plausible for weakfish, it is most likely a consequence of the von Bertalanffy growth equation imposing a negative correlation between *L_∞_* and *k* (Hesler & Lai, 2004). Similarly, slightly narrower credible intervals for measurement-error models were most likely an artifact of prior constraints on posterior values, so as to coerce biologically meaningful patterns for weakfish growth. Typically, Bayesian EIV regression can better approximate uncertainty in parameter estimates with respect to variation in both the response (i.e., length) and predictor (i.e., age). In this instance, credible intervals for posterior estimates of *L_∞_* and *k* were lessened, as estimators consistently proposed values for *L_∞_* near the upper boundary of the prior, reflecting perceived increases in asymptotic size as a consequence of incorporating ageing error during nonlinear growth curve analysis.

Measurement-error growth models can account for variability in age determinations, thereby resulting in lower estimates for variability in predicted lengths. Consequently, variance in predicted lengths appears to be overestimated if ageing error is not considered when fitting nonlinear growth curves (Table 4; Fig. 4), as the model is using discrepancies associated with age to amplify variability in predicted lengths (Fig. 4). If age reads are in any way biased or correlated, measurement-error growth curves will be unable to attenuate ageing error without validation data (i.e., reference collection), in which age for a subset of individuals is known (Punt et al., 2008). As such, it is recommended that otolith-estimated ages be used in conjunction with measurement-error growth models, as scales tend to negatively bias age estimates (Lowerre-Barbieri, Chittenden & Jones, 1994) and otolith-estimated ages tend to be more precise (Fig. 4).

The Bayesian EIV approach avoids several issues associated with previous methods to account for measurement error in age estimates during nonlinear growth curve estimation. First, it avoids uncertainty in the specification of an error variance ratio necessary for EIV functional regression as proposed by Kimura (2000). Second, the Bayesian EIV approach allows for greater flexibility in modeling ageing uncertainty and can alleviate issues with calculating a coefficient of variation for ageing error when age-length data only constitute a single age read per individual (Cope & Punt, 2007). Finally, estimation of growth curve parameters, while simultaneously considering measurement error, may improve model goodness-of-fit compared to the external, prior adjustment of observed ages before estimating regression coefficients (Spielgelhalter et al., 1996; Schwarz & Runge, 2009).

In this particular example, there is little practical difference between traditional and measurement-error von Bertalanffy growth curves (Fig. 5). In assessments of fish growth, however, we are often interested in unbiased parameter estimates that describe the underlying age-length relationship. Measurement-error models allow for more accurate estimation of parameter values (Table 4), and in this case produced estimates that are more precise (Table 2; Fig. 4). In addition, measurement-error growth models allow for estimation of the ageing error variance when multiple age determinations are unavailable, although estimation of ageing uncertainty can be improved upon when multiple age reads are available (Table 4). Estimates of the ageing uncertainty can then be used to correct for age misclassification in age-structured stock assessment models. Future research should focus on simulations that investigate the merits of using measurement-error growth models under various life histories and ageing error scenarios, as well as performance of model selection criteria in EIV contexts.

Adjustment for measurement error during model fitting is imperative, as growth models are often used to assess the relative effects of environmental factors on size (Jiao et al., 2010). By using a Bayesian EIV approach, the correlation between growth and environmental stochasticity can be discerned by removing the degrading effects of ageing error on the underlying age-length relationship. This becomes increasingly pertinent as more and more management agencies take a holistic approach to the conservation of commercial and recreational fisheries, with need to determine driving factors behind spatiotemporal trends in fish growth and productivity. Similarly, per-recruit models and the biological reference points derived from these methods are highly susceptible to variations in growth caused by ageing error (Tyler, Beamish & McFarlane, 1989), which could potentially cause overexploitation of commercially viable fish stocks and eventually lead to fishery collapse. Bayesian EIV models, then, provide a comprehensive and flexible framework upon which measurement error in observed ages can be quantified and adjusted for during model fitting, so that more accurate descriptions of growth can be used in fisheries stock assessements.

## Additional Information and Declarations

### Data Deposition

The following information was supplied regarding data availability: The data is owned by the Atlantic States Marine Fisheries Commission (ASMFC) which released the data for academic use and review only, not for publication. Access to the data can be obtained by submitting an inquiry to the Director of the Fisheries Science Program of the ASMFC.
